# Screening of *Aeromonas hydrophila* W-02 with High Polysaccharide Hydrolases Activity and Its Application in Alginate Preparation

**DOI:** 10.3390/microorganisms14071407

**Published:** 2026-06-25

**Authors:** Lijiao Wang, Chao Wang, Wenjun Deng, Qunqun Guo, Guicai Du, Ronggui Li

**Affiliations:** College of Life Sciences, Qingdao University, Qingdao 266071, China; wanglijiao@qdu.edu.cn (L.W.); wangchao6903199@qdu.edu.cn (C.W.); dengwenjun@qdu.edu.cn (W.D.); gqunqun@163.com (Q.G.)

**Keywords:** seaweed, *Aeromonas hydrophila* W-02, alginate, fiber

## Abstract

Alginate is the main structural polysaccharide in brown algal cell walls and is widely used in pharmaceuticals, textiles, cosmetics, feed, and other fields. Its tradition production methods feature low yield, high water consumption and environmental pollution, which need industrial upgrading. In this study, *Aeromonas hydrophila* W-02, a bacterial strain capable of simultaneously producing high levels of cellulase, pectinase, and glucanase, was isolated and identified from kelp humic liquid. Genome sequencing revealed that it has 5781 genes, 116 of which are related to glycoside hydrolysis. When *A. hydrophila* W-02 was applied to kelp for alginate extraction, the optimized bacterial method achieved a 38.21% yield, four times that of the traditional acid coagulation–acidification method (8.12%). Alginate was used to prepare alginate fiber and its performance indices were compared with those of commercial fibers. Scanning electron microscopy and mechanical properties analysis revealed no significant difference between alginate fibers from this novel process and commercial alginate. This study provides an efficient and green approach for alginate preparation and provides data to support the industrialization of high-efficiency bio-based alginate extraction.

## 1. Introduction

Alginate is one of the most important structural polysaccharides in brown algae. Owing to its excellent thickening capacity, versatile film-forming ability, inherent biocompatibility, and natural biodegradability, it has been widely applied in fields of food, pharmaceuticals, cosmetics and biomaterials [1,2,3]. With the rapidly growing demand for high-value-added marine polysaccharides globally, the development of efficient, green, and low-damage extraction methods for alginate from natural seaweeds has risen to the forefront of both scientific and industrial research [4,5].

Although traditional chemical extraction methods involving harsh acidic or alkaline condition are well established and widely employed industrially, they are plagued by many significant drawbacks such as polysaccharide degradation, residual impurities, and environmental concerns [6,7]. In recent years, physical assistance techniques such as ultrasonication and microwave treatment have improved the extraction efficiency, reduced extraction time, and minimized structural damage to alginate compared to traditional chemical approaches. However, there are still limitations in terms of selectivity and the preservation of biological activity [8,9].

In contrast, biological extraction methods are considered a more promising alternative strategy due to their mild reaction conditions, strong controllability, and environmental friendliness [10,11]. Despite these advantages, the widespread adoption of biological extraction methods is limited by two key factors, efficient alginate lyase-producing bacterial strains and the underlying mechanisms governing the degradation of algal substrates by microorganisms [12,13].

Based on the research background, the present study aimed to isolate and identify polysaccharides hydrolase-producing strains used for high-yield alginate from humified algal mucus, and evaluated their application potential in alginate extraction, which will provide a solid theoretical basis and practical technical support for the green preparation of alginate to meet the growing demands of various industries.

## 2. Materials and Methods

### 2.1. Materials

The kelp (*Laminaria japonica*) was purchased from the Maidao Farmers’ Market in Qingdao, China, and the specimen was kept in Herbarium of Qingdao University with a serial number of QDU-20230910-01. Dried kelp powder from the fresh kelp was prepared by dehydrating in a forced-air drying oven at 40 °C and pulverizing followed by passing through a 200-mesh standard sieve. The β-glucan, sodium carboxymethyl cellulose and glucose were purchased from Macklin Biochemical Co., Ltd. (Shanghai, China).

### 2.2. Isolation and Identification of a Multi-Enzyme Producing Bacterial Strains

Bacterial strains were enriched from humified seaweed mucilage, diluted gradiently, and plated on selective media containing β-glucan, cellulose, or pectin as the sole carbon source. After incubation at 37 °C for 48 h, strains with D/d ≥ 5 were isolated and purified by repeated streaking and re-screened by spot inoculation. Then strains showing consistently high hydrolytic activity on all three media were selected for morphological, physiological and biochemical analyses [14]. The 16S rRNA gene was amplified from genomic DNA (extracted via E.Z.N.A.^®^ Bacterial DNA Kit, Omega Bio-tek, Norcross, GA, USA) using primers 27F/1492R. Following 1% agarose gel electrophoresis, the target amplicons were recovered using the TIANgel Midi Purification Kit (Tiangen Biotech, Beijing, China) and subjected to Sanger sequencing (Sangon Biotech, Shanghai, China). In order to better understand and compare the characteristics of this strain, a phylogenetic tree was constructed using the Neighbor-Joining method in MEGA software (version 12), following the procedure described by Ying [15].

### 2.3. Further Screening of Bacterial Strains High-Yielding Intracellular Polysaccharide Hydrolases

The isolated and identified bacterial strains were subjected to 2 L fermentation. Following fermentation, the cells were disrupted by ultrasonication, and the lysate was centrifuged at 10,000× *g* for 20 min to collect the supernatant. The activities of pectinase, cellulase and β-glucanase in the supernatant were measured using the DNS method [16]. Enzyme activity is defined as the amount of enzyme required to catalyze the hydrolysis of the substrate to produce 1 μmol of reducing sugar per minute per milliliter of enzyme solution under specified conditions. Based on these enzymatic activity assays, the optimal strain demonstrating the highest overall enzyme production was selected for further investigation.

### 2.4. Whole-Genome Sequencing of the Aeromonas hydrophila W-02

Total genomic DNA was isolated from *A. hydrophila* W-02 (2 g wet weight) via the STE method [17], with quality control performed by agarose gel electrophoresis and Qubit quantification. Libraries were constructed using the NEBNext^®^ Ultra™ DNA Library Prep Kit (NEB, Ipswich, MA, USA) after DNA was sheared to 350 bp by Covaris. The process included end-repair, adapter ligation, and PCR amplification. Following initial quantification (Qubit 2.0, Thermo Fisher Scientific, Waltham, MA, USA) and size validation (Agilent 2100, Agilent Technologies, Santa Clara, CA, USA), libraries were precisely quantified by qPCR and sequenced at Novogene Co., Ltd. (Beijing, China).

Initial genome assembly was performed using Canu (Version 2.0) on the website https://github.com/marbl/canu (accessed on 25 April 2025) to generate the raw contigs. To ensure high-quality consensus sequences, the assembly underwent three successive iterations of polishing with Racon (Version 1.4.13) on the website https://github.com/isovic/racon (accessed on 25 April 2025) using the long reads, followed by three additional rounds of error correction with Pilon (Version 1.22) on the website https://github.com/broadinstitute/pilon/releases/tag/v1.22 (accessed on 25 April 2025) [18].

Protein-coding genes were predicted using GeneMarkS (Version 4.17) on the website http://topaz.gatech.edu/GeneMark (accessed on 25 April 2025) [19]. Functional annotation was conducted by aligning predicted protein sequences against functional databases using Diamond (e-value ≤ 1 × 10^−5^). The results were filtered according to the following criteria: identity ≥ 40%, coverage ≥ 40%, and only the top-scoring hits were retained. Interspersed repeats were identified using RepeatMasker (Version open-4.0.5) on the website http://www.repeatmasker.org (accessed on 25 April 2025), and tandem repeats were localized via Tandem Repeats Finder (Version 4.07b) on the website http://tandem.bu.edu/trf/trf.html (accessed on 25 April 2025).

Non-coding RNAs (ncRNAs) were further characterized as follows: tRNAs were predicted using tRNAscan-SE (Version 1.3.1); rRNAs were identified using rRNAmmer (Version 1.2) supplemented by reference sequences from closely related species; sRNAs were annotated against the Rfam database on the website https://rfam.org (accessed on 25 April 2025) and analyzed for secondary structures using the cmsearch program (Version 1.1rc4) [20].

### 2.5. Optimization of Alginate Preparation Process

To evaluate the effects of bacterial strain inoculum size and fermentation time on alginate yield, 10 g of seaweed powder was mixed with varying volumes (15, 30, and 50 mL) of *A. hydrophila* W-02 suspension (OD_600_ = 0.8). After soaking for 1 h, the mixtures were fermented in a shaking incubator at 37 °C and 170 rpm for 1, 2, and 3 days, respectively. Following fermentation, alginate was extracted from the resulting broth using two distinct methods, namely undigested approach and digested approach.

For the digested group, the fermented broth was first treated with 200 mL of 1.5% Na_2_CO_3_ solution (*w*/*v*) and incubated at 60 °C for 3 h. Subsequently, 600 mL of distilled water was added to the mixture, stirred for 2 h, and centrifuged at 10,000 rpm for 15 min. The collected supernatant was acidified to pH 2.0–3.0 with dilute HCl and allowed to stand overnight to facilitate alginic acid precipitation. The resulting precipitate was washed with distilled water to neutrality and dried to obtain the final alginate product [21].

For the undigested group, the fermented broth was directly diluted with 600 mL of distilled water without the treatment of Na_2_CO_3_ digestion, followed by the identical stirring, centrifugation, acid precipitation, and washing procedures described above.

The acid precipitation method, which is widely applied in traditional alginate production, was used as a traditional control to evaluate the extraction yield of alginate [22].

The carbazole-sulfuric acid method was used to determine the yield of alginic acid [23]. A standard curve for quantitative analysis of alginic acid was plotted with sodium alginate concentration as the abscissa and OD_530_ as the ordinate. The dried alginic acid sample was completely dissolved in distilled water, and an appropriate volume of the solution was mixed with sulfuric acid and carbazole reagent for reaction. After the reaction, the absorbance was measured at a wavelength of 530 nm. The absorbance value was then substituted into the standard curve to calculate the alginic acid concentration in the sample solution. The final yield was expressed as the grams of alginic acid actually extracted per 100 g of dry seaweed powder.

### 2.6. Preparation of Alginate Fibers and Their Microstructure

A 4% sodium alginate solution was degassed by centrifugation at 2000 rpm for 10 min at room temperature. Wet-spinning was performed using a self-assembled spinning device equipped with a single-hole spinneret. At room temperature, the spinning solution was extruded at 0.5 mL/min into a coagulation bath containing 4% CaCl_2_. The resulting calcium alginate fibers were collected by winding onto a rotating drum, thoroughly washed with deionized water to remove residual CaCl_2_, and dried at room temperature for 12 h to obtain the final alginate fibers [24].

The axial morphology of the fibers was examined by scanning electron microscopy (SEM). The fibers were mounted on conductive adhesive tape affixed to silicon wafers, sputter-coated with gold for 180 s, and observed under SEM [25].

### 2.7. Water Absorption Test of Alginate Fiber

Dried fiber samples were immersed in deionized water at room temperature until water absorption equilibrium was achieved. After removal, surface water was gently blotted with filter paper, and the samples were weighed and their diameters measured. The fully swollen fibers were transferred into centrifuge tubes fitted with a filter mesh and centrifuged at 1500× *g* for 15 min, after which the wet mass was promptly recorded. The samples were then dried at 105 °C to constant weight, cooled to room temperature, and weighed to determine the absolute dry mass. Water retention was calculated based on the wet mass and dry mass of the fibers [26].

### 2.8. Mechanical Properties Testing of Alginate Fiber

Prior to mechanical testing, all fiber samples were conditioned at 25 °C and 45% relative humidity for 2 h. Fiber diameters were measured using an optical microscope at a minimum of eight distinct positions along each sample, and the mean diameter was calculated. The stress–strain curves of the alginate fibers were acquired using an Instron 3382 universal testing machine (Instron, Norwood, MA, USA) operated at a constant crosshead speed of 20 mm/min with an initial gauge length of 20 mm [27].

### 2.9. Fourier-Transform Infrared Spectroscopy (FTIR)

The Fourier transform infrared (FTIR) spectra of the alginate samples were recorded using an FTIR spectrophotometer (Nicolet iS50, Thermo Fisher Scientific, Waltham, MA, USA) equipped with an attenuated total reflectance (ATR) accessory containing a diamond crystal. The dry powder samples were placed directly onto the ATR crystal. All spectra were collected in the wavenumber range of 4000–400 cm^−1^ with a resolution of 4 cm^−1^ and 16 co-added scans [28].

## 3. Results

### 3.1. Primary Screening of Strains

**Figure 1:**
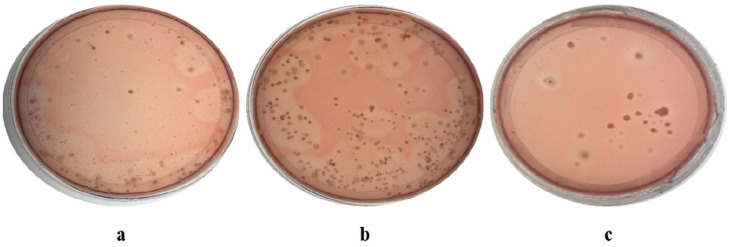
Screening of polysaccharide-degrading bacterial strains in kelp humic liquid. (**a**) β-glucan screening medium; (**b**) cellulose screening medium; (**c**) pectin screening medium.

Naturally decomposed liquid of kelp was diluted with sterilized water and plated on β-glucan, cellulose, and pectin-based media (Figure 1), five isolates (P-02, W-02, W-04, G-02, and G-04) consistently formed clear hydrolysis zones on all substrates, indicating broad polysaccharide-degrading activity.

### 3.2. Strain Identification

After incubation at 37 °C for 12 h, colony morphology was recorded. All five strains formed circular, smooth, and opaque colonies. Strain G-04 was milky white, while the other strains were beige. Microscopic examination showed that all strains were rod-shaped. Gram staining indicated that all five strains were Gram-negative. Stab inoculation tests showed that except for the G-04 strain, which had no motility, the other four strains exhibited strong motility. Flagella staining also revealed that G-04 lacked flagella, whereas the other four strains each possessed a single flagellum.

The physiological and biochemical test results of the five bacterial strains were shown in Table 1. Strains P-02, W-02, W-04, and G-02 all possessed the ability to decompose fats, starch, gelatin, and urea; whereas strain G-04 only had the ability to decompose fats and lacked the ability to decompose starch, gelatin, and urea.

**Figure 2:**
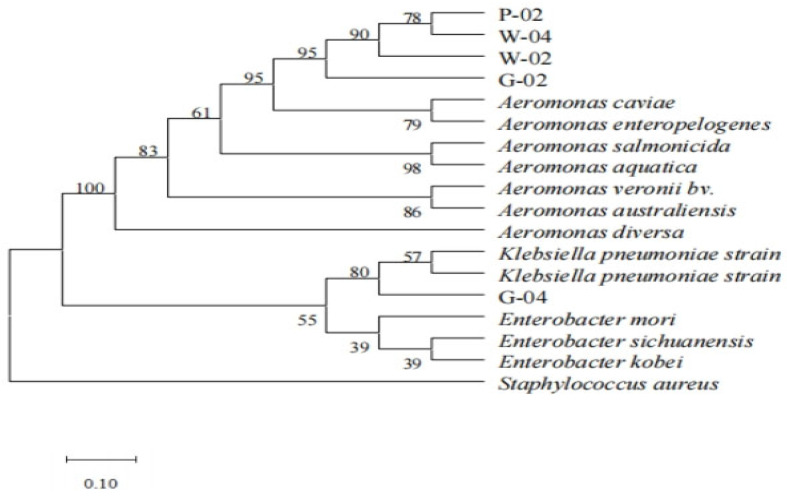
Phylogenetic tree constructed based on 16S rDNA results. Support values of bootstrap > 50% are indicated at nodes.

Using bacterial genomic DNA as the template, 16S rDNA sequences were amplified by PCR, followed by sequencing and phylogenetic analysis. Combined with the morphological characteristics and physiological and biochemical identification results of the strains, strains P-02 and W-04 were ultimately identified as *Aeromonas caviae*, and named *A. caviae* P-02 and *A. caviae* W-04, respectively. Strain W-02 was identified as *Aeromonas hydrophila* and named *A. hydrophila* W-02. Strain G-02 was confirmed to be *Aeromonas dhakensis* and designated as *A. dhakensis* G-02, while strain G-04 was identified as *Klebsiella pneumoniae* and named *K. pneumoniae* G-04 (Figure 2). The five strains were deposited at −80 °C in the Microbial Culture Collection Center of Qingdao University, assigned accession numbers as follows: P-02 (QDU-20240601), W-02 (QDU-20240602), W-04 (QDU-20240603), G-02 (QDU-20240604) and G-04 (QDU-20240605).

### 3.3. Further Screen Strains with High Intracellular Production of Polysaccharide Hydrolases

**Figure 3:**
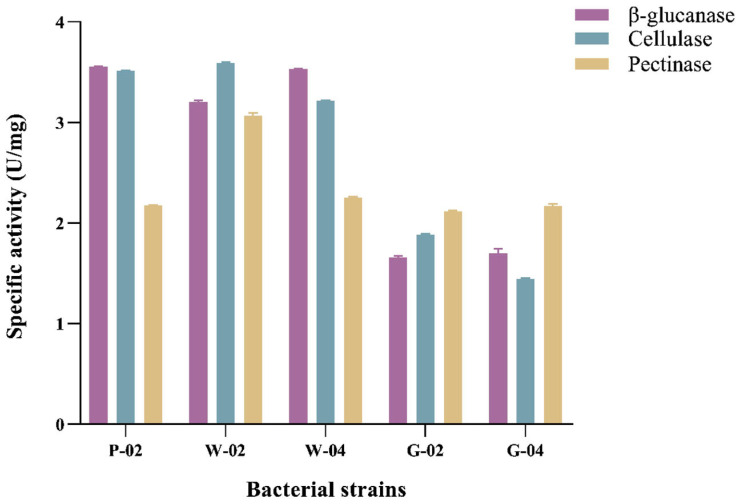
Detection of enzyme activity of different bacterial strains.

Although the five strains P-02, W-02, W-04, G-02, and G-04 all produced glucanase, cellulase, and pectinase, their extracellular enzyme concentrations were low, limiting their direct preparation from fermentation broth. Therefore, intracellular enzyme activities were determined. As shown in Figure 3, strains P-02, W-02, and W-04 exhibited high specific activities of intracellular glucanase, cellulase, and pectinase, with W-02 showing the highest activity for all three enzymes.

### 3.4. Whole-Genome Sequencing of the A. hydrophila W-02

For *A. hydrophila* W-02, the assembled genome had a total sequenced length of 4.63 Mb with a GC content of 61.48%. The minimum and maximum sequence lengths were 74 bp and 269,748 bp, respectively, with an N50 of 14,573 bp and an N90 of 10,904 bp. Gene prediction identified 5781 protein-coding genes, with a total gene length of 3.86 Mb, accounting for 83.38% of the genome. The average gene length was 668 bp. Detailed statistics are shown in Table 2.

**Figure 4:**
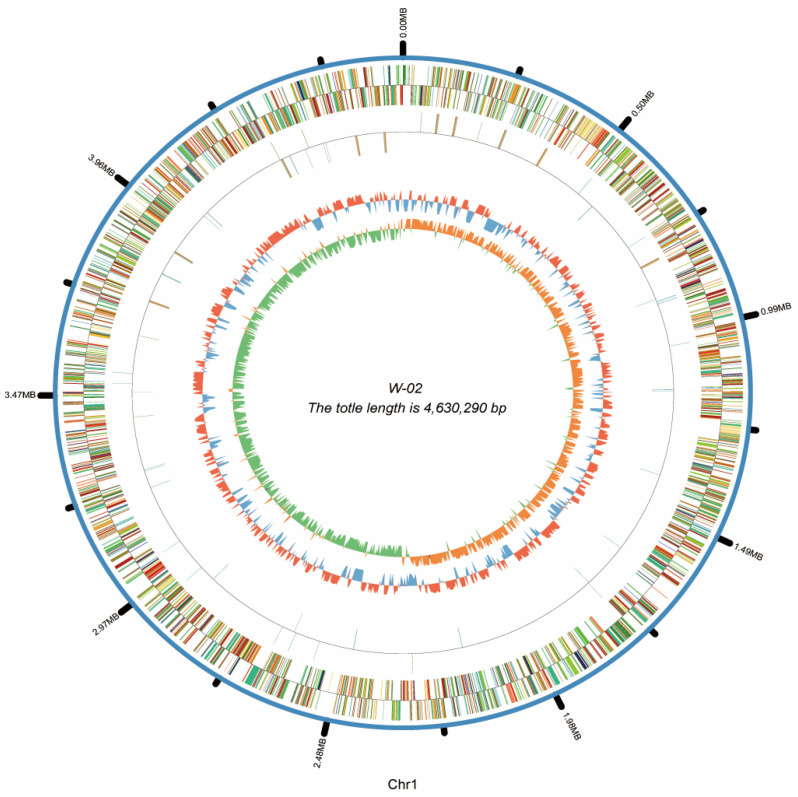
Circular diagram of the *Aeromonas hydrophila* W-02 genome.

The circular genome map provides a concise overview of the genomic features of *A. hydrophila* W-02 (Figure 4). From the outermost to the innermost circle, the tracks displayed the genome size, protein-coding genes on the forward and reverse strands annotated by the COG database, non-coding RNAs on both strands, repeat sequences, GC content, and GC skew.

A total of 9758 bp repeat sequences were identified in the *A. hydrophila* W-02 genome, including 0.22% interspersed repeats and 0.90% tandem repeats. Interspersed repeats comprised 75 retroelements (0.18% of the genome) and 13 DNA transposons (0.04%), with long terminal repeat (LTR) elements being the most abundant retroelements (0.13%). Tandem repeats were dominated by minisatellite DNA (87 elements, 0.10%) and microsatellite DNA (7 elements, 0.01%). Detailed statistics are shown in Table 3.

The non-coding RNAs included 122 tRNAs, 31 rRNAs, and 7 sRNAs, which might be involved in regulation and collectively supported basic transcriptional and translational functions, Detailed statistics are shown in Table 4.

**Figure 5:**
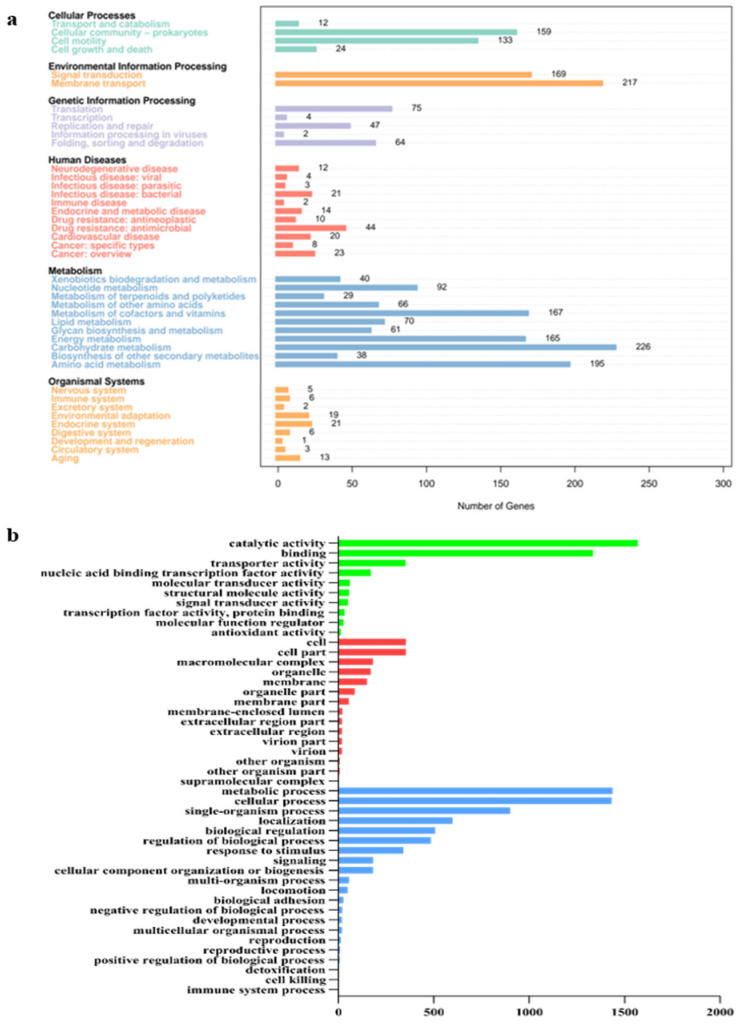
Kyoto Encyclopedia of Genes and Genomes (KEGG) and Gene Ontology (GO) functional annotation of the *A. hydrophila* W-02 genome. (**a**) Distribution of KEGG functional annotations in the *A. hydrophila* W-02 genome. The vertical axis indicates KEGG pathway categories, and different colors represent different functional classifications. (**b**) Distribution of GO functional annotations in the *A. hydrophila* W-02 genome. The horizontal axis represents the number or proportion of genes annotated to each GO term, indicating the representation of specific functions or processes in the genome. The vertical axis lists the assigned GO terms, grouped into the three main GO domains: Biological Process, Molecular Function, and Cellular Component, with further subdivision into more specific terms where applicable.

The Kyoto Encyclopedia of Genes and Genomes (KEGG) database was used for functional annotation of the *A. hydrophila* W-02 genome (Figure 5a) to elucidate the metabolic basis of its polysaccharide-degrading ability. According to KEGG pathway classification, 226 genes were involved in carbohydrate metabolism-related pathways, with 44 genes participating in the core glycolysis/gluconeogenesis pathway.

The Gene Ontology (GO) annotation of the *A. hydrophila* W-02 genome indicated that annotated genes mainly enriched in molecular functions, with 169 genes involved in transcription regulation (Figure 5b). A total of 44 genes were associated with carbohydrate metabolism and transport, covering core GO terms including GO:0005975 (carbohydrate metabolic process) and GO:0008643 (carbohydrate transport), confirming the genetic basis of the strain’s polysaccharide-degrading ability.

**Figure 6:**
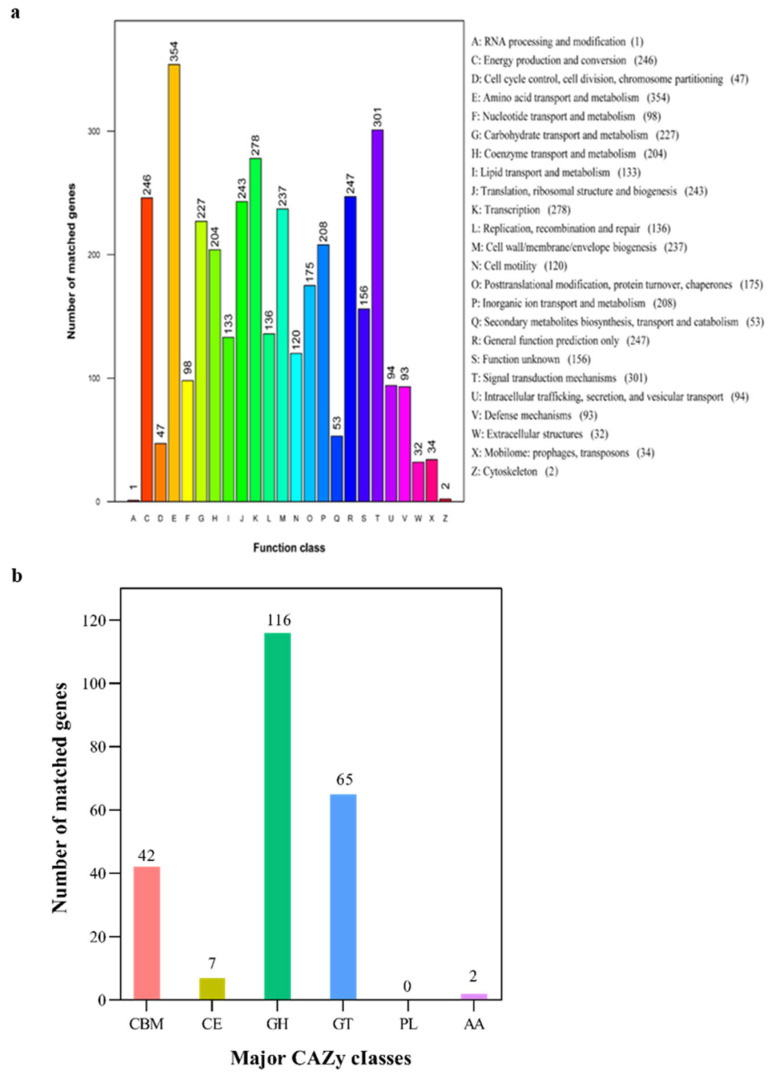
Clusters of Orthologous Groups (COG) (**a**) and Carbohydrate-Active enZymes (CAZy) functional annotation (**b**) of the *A. hydrophila* W-02 genome. (**a**) Distribution of COG functional categories in the *A. hydrophila* W-02 genome. The vertical axis indicates the number of genes assigned to each COG category, and the horizontal axis shows the different functional categories. (**b**) Distribution of CAZy families in the *A. hydrophila* W-02 genome. The vertical axis indicates the number of genes annotated to each CAZy family, while the horizontal axis lists the major CAZy classes, including glycoside hydrolases (GHs), glycosyltransferases (GTs), polysaccharide lyases (PLs), carbohydrate esterases (CEs), auxiliary activities (AAs), and carbohydrate-binding modules (CBMs).

To investigate the polysaccharide-degrading genetic basis of *A. hydrophila* W-02, COG database annotation was performed, and 3300 annotated genes were identified (Figure 6a). Functional analysis revealed that 394 genes (11.93%) were associated with amino acid transport and metabolism, while 227 genes (6.88%) were related to carbohydrate transport and metabolism. The identified key carbohydrate-active genes included COG2723 (β-glucosidase), COG3405 (glucanase), and COG1472 (periplasmic beta-glucosidase). The abundance of carbohydrate metabolism and transport-related genes confirms that *A. hydrophila* W-02 has a solid genetic foundation for polysaccharide degradation.

A total of 232 carbohydrate enzyme-related genes were identified through Carbohydrate-Active Enzymes (CAZy) database annotation of the *A. hydrophila* W-02 genome. The glycoside hydrolase (GH) family was the most abundant with 116 genes, followed by the glycosyltransferase (GT) family with 65 genes, carbohydrate-binding module (CBM) family with 42 genes, carbohydrate esterase (CE) family with 7 genes, and auxiliary activity (AA) family with 2 genes. The proteins encoded by these genes included glucosidase mainly belonging to the GH1, GH2, GH3, and GH5 families, endoglucanase (EC 3.2.1.4) mainly belonging to the GH6 and GH9 families, and α-glucosidase (EC 3.2.1.20) mainly belonging to the GH13 and GH63 families. The presence of these genes enables the strain to decompose polysaccharides efficiently, and confirms the polysaccharide-degrading ability of *A. hydrophila* W-02 at the genetic level (Figure 6b).

### 3.5. Investigation on the Extraction Process of Alginate

**Figure 7:**
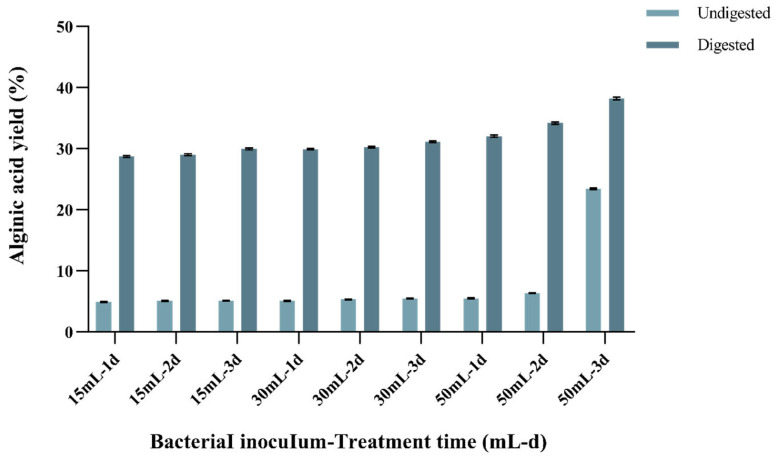
Effect of inoculum dosage and fermentation time on alginate yield.

The effects of inoculum volume and fermentation time were evaluated. Within the testing range, both parameters had a positive impact on alginate yield, with the highest yields obtained at 50 mL inoculum and 3 days of fermentation: 38.21% in the digestion group and 23.51% in the non-alkaline digestion group (Figure 7).

**Figure 8:**
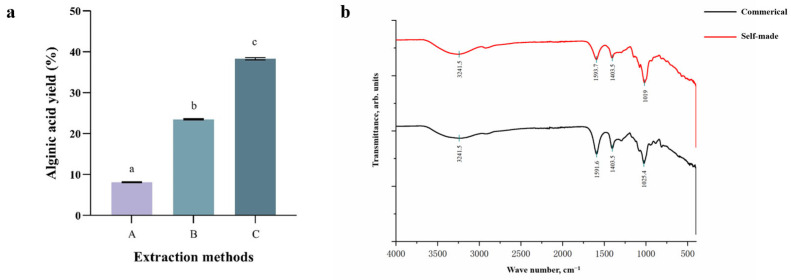
Extraction and FTIR analysis of alginate. (**a**) Effects of different extraction methods on alginate yield. A, Traditional method; B, Bacterial method (without digestion); C, Bacterial method (with digestion). Note: a, b, c mean significant differences at *p* < 0.05. (**b**) FTIR analysis of alginates.

As shown in Figure 8a, the integrated bacterial method produced higher alginate yields than individual bacterial or enzymatic method. Without alkali digestion, the yields were 23.51%, while after digestion, the yield increased to 38.21%. In contrast, the alginate yield in the traditional acid precipitation method was only 8.12%. Therefore, compared with traditional methods, the integrated method achieved a 2.8-fold increase without alkali digestion and a 4.7-fold increase with alkali digestion.

FTIR spectrum of the self-made alginate exhibited an absorption profile similar to the commercial alginate. In both spectra, the broad band observed at 3241.5 cm^−1^ was attributed to the O–H stretching vibrations. The characteristic peaks corresponding to the asymmetric and symmetric stretching vibrations of the -COO^−^ groups were observed at 1591.6 cm^−1^ and 1403.5 cm^−1^ for the commercial alginate, and at 1593.7 cm^−1^ and 1403.5 cm^−1^ for the self-made sample, respectively. Furthermore, the absorption bands at 1025.4 cm^−1^ (commercial) and 1019 cm^−1^ (self-made) were assigned to the C–O–C and C–O stretching vibrations of the pyranose ring [29].

### 3.6. Preparation and Microscopic Morphology of Alginate Fibers

**Figure 9:**
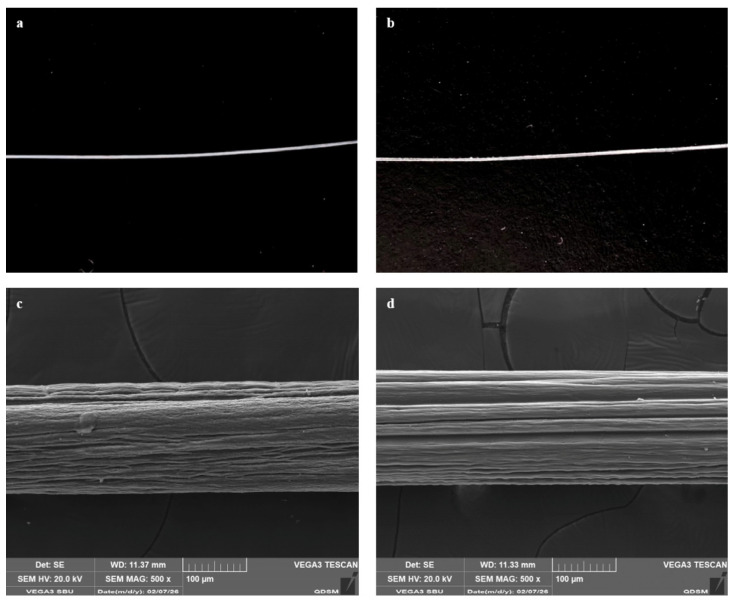
Alginate fiber sample under light microscope (LM) and scanning electron microscope (SEM). (**a**) Self-made alginate fiber under LM; (**b**) Commercial alginate fiber under LM; (**c**) Surface image of self-made alginate fiber under SEM; (**d**) Surface image of commercial alginate fiber under SEM.

As shown in Figure 9, the alginate prepared in this study was successfully spun into continuous and intact alginate fibers. Their macroscopic morphology and microstructure were comparable to fibers prepared from commercial alginate. These results demonstrated that the prepared alginate has good spinnability and comparable performance to commercial products.

### 3.7. Water Absorption Test of Alginate Fiber

As shown in Table 5, the self-made alginate fibers showed comparable liquid absorption, water retention, and swelling properties to commercial alginate fibers, with values of 12.9 ± 0.17 g/g, 801 ± 5.4%, and 211 ± 0.8 μm, respectively, indicating similar moisture absorption and retention performance.

### 3.8. Mechanical Properties Testing of Alginate Fiber

**Figure 10:**
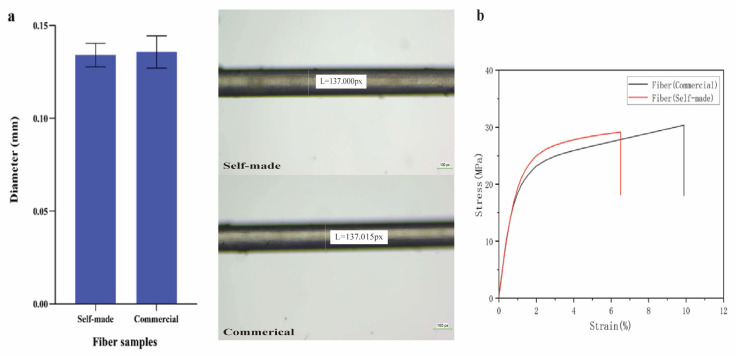
Diameter and mechanical properties of self-made and commercial fibers. (**a**) Comparison of fiber diameter between self-made and commercial fibers, together with representative optical images. Error bars represent standard deviation; (**b**) Stress–strain curves of self-made and commercial fibers.

As shown in Figure 10a, the self-made and commercial fibers showed comparable diameters and similar morphological features. The tensile test revealed that the stress–strain profile of self-made fibers was slightly higher than that of the commercial fibers (Figure 10b). Both fibers achieved a maximum stress of approximately 30 MPa and comparable breaking strains, suggesting similar mechanical performance. These findings indicated that the self-made fibers have slightly enhanced tensile properties and promising application potential.

## 4. Discussion

This study successfully isolated five efficient algal polysaccharide-degrading strains from humified kelp using a composite-carbon-source screening strategy. Subsequent measurement of intracellular β-glucanase, cellulase, and pectinase activities showed that *A. hydrophila* W-02 exhibited the highest specific activity for all three enzymes, making it the most promising candidate for further study. These specific enzymes must work synergistically to break down kelp, because the tough cell wall matrix of kelp is made up of an interwoven network of cellulose, hemicellulose, and pectin-like substances that tightly wrap alginate [30]. This finding is consistent with previous reports that *A. hydrophila* can naturally produce cellulase and pectinase and possesses intrinsic polysaccharide-degrading capability [31,32]. Moreover, the strong multi-enzyme activity observed in W-02 further extends the enzymatic profile of *A. hydrophila* and suggests its potential to degrade complex algal cell wall polysaccharides.

The genomic architecture of *A. hydrophila* W-02 (4.63 Mb, 61.48% GC) showed high synteny with the type strain ATCC 7966T, confirming its taxonomic identity and high-quality the assembly [33]. Although *A. hydrophila* W-02 shares high genomic synteny with the type strain ATCC 7966T, it lacks the typical major high-pathogenicity markers of clinical isolates. This indicated a shift in its evolution towards a saprophytic lifestyle in special environments with higher biosafety.

Functional annotation reveals a clear divergence in metabolic specialization between *A. hydrophila* W-02 and its pathogenic counterparts, such as strains XDMG, HX-3, and the type strain ATCC 7966T. While these reference strains are characterized by extensive virulence factors and quorum-sensing (QS) systems (e.g., AI-1/2/3 in XDMG and AHL signaling in HX-3) tailored for host infection [33,34,35], W-02 exhibits a significantly expanded genetic repertoire dedicated to carbohydrate utilization. Notably, the number of genes in COG category G (Carbohydrate transport and metabolism) in W-02 (249 genes) far exceeds that in XDMG (181 genes), indicating a more robust system for the uptake and processing of diverse sugars.

This metabolic bias is further evidenced by the KEGG and CAZy profiles. W-02 possesses a superior capacity for glycan biosynthesis and metabolism (100 genes) compared with HX-3 and XDMG, suggesting a higher efficiency in secreting polysaccharide hydrolases. More importantly, the abundance of 116 GHs and 42 CBMs in W-02 underscores its specialized ability to degrade complex algal biomass, while the genomes of ATCC 7966T and HX-3 focused more on environmental resistance and host–pathogen interactions. Such functional enrichment reflects the evolutionary adaptation of W-02 to the polysaccharide-rich environment of humified kelp, transforming the versatile *A. hydrophila* scaffold into a high-performance biocatalyst for algal biomass refining. Numerous studies have demonstrated that microorganisms isolated from algal habitats generally encode abundant carbohydrate-active enzymes, including diverse alginate lyases, to efficiently utilize algal polysaccharides. A recent characterization of a novel endolytic alginate lyase belonging to the PL15 family further confirms that algae-derived bacteria rely on functional polysaccharide-degrading enzymes to adapt to algal environments and depolymerize alginate [36]. Unlike traditional chemical methods that non-selectively attack all polysaccharides, the specific functional enrichment of strain W-02 makes it a highly targeted biocatalyst, which selectively decomposes the cellulose barrier in kelp cell walls without damaging the structural integrity of target alginate macromolecules [37].

Building on the promising genomic capabilities of W-02 in polysaccharide degradation, we further evaluated its practical potential for green biomanufacturing by directly applying it to alginate extraction from kelp, followed by fiber spinning.

This study established a potent bacterial strategy that effectively disrupts the recalcitrant seaweed matrix, and significantly enhanced alginate extraction efficiency. The integrated method achieved a 4.7-fold increase in yield (38.21%) with alkali digestion and a 2.8-fold increase (23.51%) without it, compared to the traditional acid precipitation benchmark (8.12%). Mechanistically, the high alginate yield achieved under mild conditions stems from W-02 enzymatically pre-loosening the kelp matrix, which greatly improves alginate accessibility to extraction solvents and reduces dependence on harsh chemical degradation [37]. Although previous studies have explored physical or single-enzyme pretreatments for alginate extraction [38,39], these methods often struggle to balance high yields with low chemical consumption. Here, our proposed synergistic route effectively overcomes this bottleneck. By demonstrating high efficiency under completely alkali-free conditions, this strategy offers a promising, green, and sustainable alternative to traditional heavily chemical-dependent alginate processing.

Valorization of industrial and agricultural residual biomass has become a promising direction for developing natural biopolymers. A novel functional polysaccharide has been isolated from the residues after essential oil extraction of *Alpinia zerumbet* fruits, fully demonstrating the high-value development potential of waste resources [40]. Aligned with this research concept, the present study uses humified kelp as raw material to establish a microbial-mediated alginate extraction strategy, which not only improves alginate extraction efficiency but also provides a feasible approach for comprehensive utilization of waste marine biomass.

To validate its practical applicability, the extracted alginate was processed into fibers via wet spinning. The resulting fibers exhibited a dense morphology, alongside mechanical strength and fluid-handling properties (absorbency and water retention) highly comparable to those derived from commercial alginate. Frequently, lab-extracted alginates suffer from poor gelation or structural instability due to crude processing [41,42]. Overcoming this common bottleneck, our product demonstrates superior fiber-forming performance. This indicates that the proposed bacteria process not only achieves green extraction, but also produces high-quality biopolymers directly applicable for downstream functional material manufacturing.

## 5. Conclusions

In this study, we isolated *Aeromonas hydrophila* W-02, a strain with high production of polysaccharide hydrolases from kelp humic liquid, which harbors 116 glycoside hydrolase-related genes among 5781 total genes. This strain was used for alginate production, and our biological extraction achieved a 38.21% alginate yield, 4-fold higher than that of the traditional acid method. The extracted alginate fiber showed no significant performance difference from commercial products, and this work provides an efficient green approach for industrial bio-based alginate production.

## Figures and Tables

**Table 1:** Physiological and biochemical characteristics of the five bacterial strains.

Test	P-02	W-02	W-04	G-02	G-04
Lipase	+	+	+	+	+
Amylase	+	+	+	+	−
Gelatin Hydrolysis	+	+	+	+	−
Glucose fermentation	+	+	+	+	+
Sucrose fermentation	+	+	+	+	+
Lactose fermentation	−	−	−	−	−
Urease	−	−	−	−	+
Methyl Red	+	+	+	+	−

+ indicates positive, − indicates negative.

**Table 2:** Genomic features of *Aeromonas hydrophila* W-02.

Features	Value
Total sequenced length (bp)	4,630,290
Min sequence length (bp)	74
Max sequence length (bp)	269,748
N50	14,573
N90	10,904
GC content %	61.48
Total genes length (bp)	3,860,646
Genes percentage of genome	83.38%
Total genes number	5781
Average gene length (bp)	668

**Table 3:** Statistical analysis of repeat sequences in the *A. hydrophila* W-02 genome.

Repeat Type	Number of Elements	Total Length (bp)	In Genome (%)
LTR	47	5885	0.1271
DNA	13	1811	0.0391
LINE	15	1199	0.0259
SINE	12	1070	0.0231
RC	1	79	0.0017
Tandem repeats	173	41,849	0.9038
Minisatellite DNA	87	4804	0.1038
Microsatellite DNA	7	331	0.0071

**Table 4:** Statistical analysis of ncRNA in the *A. hydrophila* W-02 genome.

Type	Number	Average Length (bp)	Total Length (bp)
tRNA	122	78	9545
5s rRNA	11	114	1255
16s rRNA	10	1537	15,367
23s rRNA	10	2892	28,923
sRNA	7	155	1087

**Table 5:** Water absorption test of alginate fibers.

Value	Self-Made Fiber	Commercial Fiber
Liquid absorption capacity (g/g)	12.9 ± 0.17	13.3 ± 0.07
Water retention rate (%)	801 ± 5.4	807 ± 7.4
Diameter swelling (μm)	211 ± 0.8	209 ± 1.7

## Data Availability

The original contributions presented in this study are included in the article. Further inquiries can be directed to the corresponding authors.
